# Eponyms in Otology: A Historical Narrative of Commonly Used Surgical Instruments

**DOI:** 10.1002/oto2.52

**Published:** 2023-05-09

**Authors:** J. Joseph Caraway, Alex W. Yang, Anthony M. Tolisano

**Affiliations:** ^1^ School of Medicine Uniformed Services University of the Health Sciences Bethesda Maryland USA; ^2^ Department of Otolaryngology–Head and Neck Surgery Walter Reed National Military Medical Center Bethesda Maryland USA; ^3^ Department of Surgery Uniformed Services University of the Health Sciences Bethesda Maryland USA

**Keywords:** eponymous instruments, otology, tympanoplasty

## Abstract

Numerous eponymous instruments are regularly used in the otology surgical suite. This manuscript uses a tympanoplasty to highlight 10 frequently used instruments and the remarkable surgeons behind their creation. Many of these names will be familiar, but we hope our readers will gain an appreciation of these landmark figures that have transformed the practice of otology.

Surgical technique is heralded for its newest innovation, yet the instrumentation is steeped in history. Eponymous instruments are an homage to the past. This paper explores the people behind select otologic instrumentation using endoscopic‐assisted tympanoplasty as its framework. A more comprehensive summary of eponymous otologic instruments is listed in Supplemental Table S1, available online.

## Endoscopic Tympanoplasty

After surgical preparation, a *Hopkins endoscope* is inserted into the ear canal. Dr Harold H. Hopkins (1918‐1994) was a British physicist who excelled in optical physics and developed modern flexible and rigid endoscopes with Karl Storz. He was twice nominated for the Nobel Prize in Physics.^1^

A *Baron suction* is used to clear the canal of debris. Dr Shirley H. Baron (1904‐1979) completed his medical degree and otolaryngology residency in New York. He was later commissioned as a United States Navy Medical Officer during WWII and served in the Pacific Theatre from 1941 to 1946. Although his expertise in otolaryngology was broad, Dr Baron was particularly recognized for his teachings regarding the management of cholesteatoma. He was co‐chair of the University of California San Francisco Department of Otolaryngology and President of the Triological Society.^2^

Next, the *Rosen pick* is used to freshen the edges of the perforation. Dr Samuel Rosen (1897‐1981) was a Jewish‐American otolaryngologist who first described stapes mobilization as a treatment for otosclerosis. He immigrated to the United States from Poland and with the financial support of his siblings, attended medical school at Syracuse University. He became a well‐trained otologic surgeon, successfully practicing the stapes fenestration technique described by Dr Julius Lempert. He would accidentally discover the stapes mobilization technique by pushing on an affixed stapes bone a millimeter too far during otosclerosis surgery, instantly restoring his patient's hearing. Despite initial denunciations as unsafe, his mobilization technique eventually gained worldwide acceptance.^3^

The tympanomeatal flap is elevated using a *House round knife* and the middle ear is entered. Dr William F. House (1923‐2012) is often referred to as “the father of neurotology” due to his groundbreaking developments in ear surgery and the invention of the cochlear implant. He was a United States Navy dental officer before leaving the military to complete medical school and residency in Los Angeles. He established the House Institute with his brother Dr Howard House to tackle problems of the inner ear. He is well known for introducing the operating microscope to neurotology/neurosurgery and popularizing presigmoid approaches to the lateral skull base, his expertise in managing Meniere's disease allowed astronaut Alan Shepard to return to space and fly to the moon.^4^

If a postauricular incision is made, a self‐retaining *Weitlaner retractor* is used to facilitate exposure. Dr Franz Weitlaner (1872‐1944) was an Austrian physician who developed the Weitlaner retractor (often misspelled as “Weitlander” or “Wheatlander”). Dr Weitlaner studied at the Innsbruck Medical University in Austria and upon completion of his training, worked as a shipboard physician with the Austrian Lloyd shipping company. He became a community physician in northeast Austria where the inspiration for the self‐retaining retractor derived from acknowledging that it was “…not unusual for a general practitioner to have to undertake small or large surgical operations without sufficient assistance.”^5^

The soft tissues are elevated from the mastoid cortex using a *Freer elevator*. Dr Otto “Tiger” Freer (1857‐1939) was an American otolaryngologist who graduated from Rush Medical College in 1879 and later specialized in laryngology at the German Hospital of Chicago from 1894 to 1905. Along with Dr Gustav Killian, he is credited with popularizing submucosal resection of the nasal septum and from these devised a new set of instruments. He initially described his innovative elevators as flat knives with rounded, paddle‐shaped blades whose original application was to correct nasal septum deflections.^6^

Temporalis fascia is harvested and pressed with the *Sheehy fascia press*. Dr James L. Sheehy (1926‐2006) was an American otologist. He received his medical degree from Stanford University in 1951 and completed his residency at Letterman Army Hospital before serving as otolaryngologist in the U.S. Army Medical Corps where he concentrated his efforts in otology. Dr Sheehy helped codify the lateral graft tympanoplasty and intact canal approach to mastoidectomy. He was also a gifted teacher and researcher, teaching extensively on surgical technique and nonsurgical issues that were often difficult to manage, such as vertigo and tinnitus.^7^

The graft is introduced into the middle ear space with the *Hartmann alligator forceps*. Dr Arthur Wilhelm Hartmann (1849‐1931) was a German otologist who developed numerous medical instruments such as the Hartmann ear forceps and nasal speculum. He served as a medical assistant in the third Württemberg Field Hospital during The Franco‐Prussian War before finishing his medical degree at the University of Leipzig in 1873. He would serve as a military doctor for 2 years before devoting himself to otology, traveling to Vienna to train under some of the leading otolaryngologists of the time. Dr Hartmann settled in Berlin and maintained a private clinic where he instructed several apprentices, some of whom like Dr Gustav Killian would become renowned otolaryngologists themselves. He produced several important medical literary works, the most popular being *Diseases of the Ear and Their Treatment* which had 8 editions and was translated into English, French, and Italian.^8^

Once the graft is in an adequate position, the tympanomeatal flap and vascular strip are returned to their anatomical position. This can be confirmed through the ear canal using the *Lempert speculum*. Dr Julius Lempert (1890−1968) was a Jewish‐American ear surgeon best known for his development of the fenestration technique for otosclerosis. He established a small hospital in Manhattan from a converted 5‐story building into a new otological medical center which he called the Endaural Hospital. With a dental drill, Dr Lempert's procedure involved the creation of a new window on the horizontal canal, allowing sound wave energy to stimulate the inner ear past an affixed stapes footplate. He never accepted newer operative methods for otosclerosis such as stapes mobilization by Dr Samuel Rosen and later stapedectomy by Dr John Shea III, both of which would eventually overtake his fenestration technique.^9^

The incision is closed, the ear is packed, and a *Glasscock dressing* is applied. Dr Michael E. Glasscock III (1933‐2018) was a distinguished neurotologist who founded the Otology Group of Vanderbilt and its neurotology fellowship, recognized worldwide as a leading practice and training ground for disorders of the ear. He trained under several pioneers in neurotology including Dr John Shea III and Dr William House. He founded the *American Journal of Otology* (what is now Otology and Neurotology) and served as President of the American Otological Society in 1992.^10^

## Author Contributions

**J. Joseph Caraway**, study design, primary research, drafting/revision of manuscript; **Alex W. Yang**, study design, primary research, drafting/revision of manuscript; **Anthony M. Tolisano**, study design, drafting/revision of manuscript.

## Disclosures

### Competing interests

The opinions or assertions contained herein are views of the authors and are not to be construed as official or reflecting the views of the Department of Defense, the Uniformed Services University of the Health Sciences, or any other agency of the U.S. Government.

### Funding source

None.

## Supporting information

A more complete listing of eponymous ear instruments.

## References

[1] Bhatt J , Jones A , Foley S , et al. Harold Horace Hopkins: a short biography. BJU Int. 2010;106(10):1425‐1428. 10.1111/j.1464-410x.2010.09717.x 21049584

[2] Boles R . Shirley H. Baron, MD, 1904‐1979. Arch Otolaryngol Head Neck Surg. 1980;106(9):591‐592. 10.1001/archotol.1980.00790330071026 6996659

[3] Bhutta M . A one‐millimeter push revolutionizes ear surgery: the story of Samuel Rosen and surgery of the stapes bone. Hektoen Int J. 2016;8(3). https://hekint.org/2017/01/30/a-one-millimeter-push-revolutionizes-ear-surgery-the-story-of-samuel-rosen-and-surgery-of-the-stapes-bone/

[4] Berliner KI . In memoriam: William F. House, D.D.S., M.D., the “father of neurotology” 1923‐2012. Otol Neurotol. 2013;34(3):386‐387. 10.1097/MAO.0b013e3182877b43 23446324

[5] Sharma A , Swan KG . Franz Weitlaner: the great spreader of surgery. J Trauma Injury Infect Crit Care. 2009;67(6):1431‐1434. 10.1097/TA.0b013e3181b2fe3e 20009698

[6] Chittiboina P , Connor Jr DE , Nanda A. Dr. Otto “Tiger” Freer: inventor and innovator. Neurosurg Focus. 2012;33(2):E12. 10.3171/2012.6.FOCUS12137 22853830

[7] Brackmann DE . In memoriam: James Sheehy: December 21, 1926‐March 22, 2006. Otol Neurotol. 2006;27(8):1051‐1052. 10.1097/01.mao.0000247809.55335.a7

[8] Zarniko C . Zu Arthur Hartmanns 80. Geburtstage. Hamburg, Archiv für Ohren‐, Nasen‐ und Kehlkopfheilkunde. Bd. 1929;120(2‐3):I‐VI.

[9] Pietruski J . [Juliusz Lempert (1890‐1959): the author of the fenestration technique]. Otolaryngologia polska = Pol Otolaryngol. 1998;52(3):341‐346. 9760779

[10] Batcheldor M . *VUMC mourns renowned neurotologist Glasscock*. Vanderbilt University. 2018. Accessed January 9, 2023. https://news.vumc.org/2018/02/22/vumc-mourns-renowned-neurotologist-glasscock/

